# Challenges in diagnosis and management of diabetes in the young

**DOI:** 10.1186/s40842-016-0036-6

**Published:** 2016-11-10

**Authors:** Ranjit Unnikrishnan, Viral N. Shah, Viswanathan Mohan

**Affiliations:** 1 Madras Diabetes Research Foundation & Dr Mohan’s Diabetes Specialties Centre, Who Collaborating Centre for Non-Communicable Diseases Prevention and Control, 4, Conran Smith Road, Gopalapuram, Chennai, 600 086 India; 2 Barbara Davis Center for Diabetes, University of Colorado Anschutz Campus, Aurora, CO USA

**Keywords:** Diabetes in youth, Type 1 diabetes, Type 2 diabetes, Childhood diabetes, Monogenic diabetes, Fibrocalculous pancreatic diabetes

## Abstract

The prevalence of diabetes in children and adolescents is increasing worldwide, with profound implications on the long-term health of individuals, societies, and nations. The diagnosis and management of diabetes in youth presents several unique challenges. Although type 1 diabetes is more common among children and adolescents, the incidence of type 2 diabetes in youth is also on the rise, particularly among certain ethnic groups. In addition, less common types of diabetes such as monogenic diabetes syndromes and diabetes secondary to pancreatopathy (in some parts of the world) need to be accurately identified to initiate the most appropriate treatment. A detailed patient history and physical examination usually provides clues to the diagnosis. However, specific laboratory and imaging tests are needed to confirm the diagnosis. The management of diabetes in children and adolescents is challenging in some cases due to age-specific issues and the more aggressive nature of the disease. Nonetheless, a patient-centered approach focusing on comprehensive risk factor reduction with the involvement of all concerned stakeholders (the patient, parents, peers and teachers) could help in ensuring the best possible level of diabetes control and prevention or delay of long-term complications.

## Background

The increasing prevalence of diabetes among adolescents and young adults below 20 years of age is, perhaps, the most worrisome aspect of the global epidemic of diabetes. Development of diabetes at young ages has several repercussions on the health of affected individuals and translates to significant morbidity and mortality, as well as the loss of economic productivity. The diagnosis and management of diabetes in these young patients presents several unique challenges, many of which may not be familiar to physicians accustomed to dealing only with middle-aged and elderly patients with diabetes. The purpose of the present review is to offer an overview of the various types of diabetes in the young and to discuss the ways and means of overcoming the challenges commonly encountered in the diagnosis and management of these patients.

### Magnitude of the problem

According to the International Diabetes Federation, there are almost 500,000 children worldwide with type 1 diabetes (T1D) as of 2013, 26 % of whom reside in Europe [1]. The prevalence of T1D appears to be slowly increasing in some countries while it is stable in others. It is clear that type 2 diabetes (T2D) among children and adolescents is also on the rise, but robust data is lacking from many parts of the world as population based screening for this disorder is currently not recommended [2]. The SEARCH for Diabetes in Youth Study estimated that as of 2009, 1 in 433 children and adolescents in the United States had diabetes, the majority having T1D and a significant minority having T2D [3]. There also appears to be ethnic variability in the susceptibility to different types of diabetes, with T1D being more common in non-Hispanic whites and T2D in ethnic groups where the background prevalence of T2D is high (Native Americans, Hispanics, and African Americans). While there is a paucity of comparative data from other parts of the world (particularly Asia), available evidence suggests that T2D is now more common than T1D as a cause of diabetes among youth in Taiwan, Japan and Hong Kong [4].

### Spectrum of diabetes in youth

The differential diagnosis of diabetes in middle and old age is usually straightforward as the majority of patients have T2D in this age group. However, the situation is different in children, adolescents and young adults where a spectrum of hyperglycemic disorders needs to be considered in the differential diagnosis. The differential diagnosis of diabetes in this age group occasionally presents significant challenges, on account of the emergence of T2D, and the increasing prevalence of obesity in the general population, which minimizes the value of body mass index as a distinguishing feature between T1D and T2D. The following section will deal with the salient features of some of the common types of diabetes in children and adolescents.

### Type 1 diabetes

T1D is characterized by profound hyperglycemia due to absolute insulin deficiency caused by immune associated destruction of the insulin-producing beta cells of the pancreas. Worldwide, the incidence and prevalence of type 1 diabetes vary substantially with high prevalence in Finland and lower rates in China, India, and Venezuela [5]. The age of onset of T1D appears to be significantly higher in Africa as compared to Europe [6].

While the incidence of T1D is highest from age 5 till adolescence, it can be diagnosed at any age. Nearly 90 % of individuals with type 1 diabetes have presence of one or more islet autoantibodies such as insulin (IAA), glutamic acid decarboxylase (GADA), insulinoma-associated autoantigen 2 (IA-2), and zinc transporter 8 (ZnT8A) [7]. These autoantibodies may be present months to years before symptomatic onset, enabling identification of at-risk individuals and interventions to possibly modify the course of disease in the future (primary or secondary prevention). However, in some parts of the world (e.g. Africa and Asia), a significant proportion of patients do test negative for autoantibodies and this type of T1D is termed idiopathic (type 1B) [8–10].

A recent consensus statement from the Juvenile Diabetes Research Foundation, American Diabetes Association and the Endocrine Society [11] has identified three stages of “early” T1D- Stage 1, where the individual has evidence of autoimmunity but is normoglycemic, Stage 2, where there is evidence of glucose intolerance, and Stage 3, characterized by symptomatic hyperglycemia. The Statement also recognizes a “Pre-Stage 1”, which includes individuals with a genetic predisposition to islet-cell autoimmunity in whom autoantibodies are as yet undetectable.

Recent studies have clearly shown that type 1 diabetes is a polygenic disorder, with nearly 40 loci known to affect disease susceptibility. Genes of the major histocompatibility complex (Human Leucocyte Antigen-HLA) are perhaps the best risk markers for the development of T1D, In Caucasians, HLA Class II antigens, particularly DR4-DQ8, are associated with a high risk of T1D; however, the exact HLA types associated with disease vary among different ethnic groups [12]. Class I MHCs also seem to influence risk for type 1 diabetes, independent of class II molecules. In addition to genetic factors, seasonal variations in the incidence of T1D supports a role of an environmental factors in pathophysiology of type 1 diabetes. The environmental factors implicated in triggering of the autoimmune response include viral and bacterial infections, dietary factors and deficiency of vitamins and nutrients [13, 14].

### Type 2 diabetes

Type 2 diabetes (T2D) represents a broad spectrum of disorders, ranging from severe insulin resistance with only minimal insulin secretory defect to profound insulin secretory defect with minimal insulin resistance. Though T2D is considered as a disease of older individuals, the prevalence of T2D in youth is higher in certain ethnic groups such as Native Americans and Pacific Island populations [15]. In Japan, more than 80 % of all new cases of diabetes in children and adolescents were diagnosed as T2D [16], while in Europe, the prevalence rates appear to be significantly lower [17]. A clinic-based study from Jamaica found that 22 % of individuals diagnosed with diabetes prior to the age of 25 years had features consistent with T2D [18]. In the Indian Council of Medical Research (ICMR) Registry of Diabetes in the Young in India, 25.3 % of individuals developing diabetes under the age of 25 years had a diagnosis of T2D [19]. The increase in prevalence of T2D is closely linked to the epidemic of obesity affecting youth in many countries across the world [20].

While genetic factors undoubtedly play a role in the development of T2D, they alone cannot explain the explosive increase in the prevalence of disease seen in the past three decades, particularly in developing countries and among youth. Increasing exposure to unhealthy “diabetogenic” environmental factors such as high intake of calorie-rich, refined foods and sedentary lifestyle may accelerate the development of diabetes in young people who are predisposed to the development of disease [21].

There is a debate as to whether insulin resistance or beta cell secretory defect represents the major pathophysiology in T2D presenting in youth. While most overweight or obese youth are insulin resistant, diabetes does not develop unless beta cell dysfunction supervenes [22]. In Asian Indian youth with diabetes, beta cell dysfunction has been found to be quantitatively more important than insulin resistance, in the development of T2D [23]. Similarly, it has been suggested that among East Asians with youth-onset diabetes, genetically determined beta-cell dysfunction predisposes to diabetes in the setting of even mild decreases in insulin sensitivity [6]. Individuals with diminished beta cell reserve are less able to cope with increased demand for insulin imposed by the development of insulin resistance, and develop hyperglycemia consequent to beta cell exhaustion. Diminution of beta cell reserve may be mediated by genetic factors, or by early life or in utero programming (brought about by in utero malnutrition, which remains a pressing problem in many parts of the world), or as a result of chronic hyperstimulation from insulin resistance in early life [22, 24]. Of interest, Asian Indians have been shown to have higher insulin resistance than their Caucasian counterparts, even from childhood [25, 26]. It has also been shown that Asian Indian neonates have higher concentrations of insulin in their cord blood even though they were, on average, smaller than Caucasian neonates [27].

### Monogenic diabetes

In contrast to T1D and T2D, monogenic diabetes is caused by defects in a single gene. Monogenic diabetes accounts for 1 to 2 % of diabetes in the young in the U.K and the USA [28, 29]. Data from other parts of the world is not as readily available, but is likely to show comparable results. In a clinic based study from India, genetic defects were found in approximately 12 % of patients identified for screening using clinical criteria [30, 31].

The first description of monogenic diabetes in mainstream literature was published by Tattersall and Fajans in 1975 [32]. They described a series of non-insulin dependent diabetes with autosomal dominant transmission in young adults, and termed it maturity onset diabetes of the young (MODY). Early age at onset (less than 25 years), insulin independence for at least 5 years from diagnosis, autosomal dominant inheritance and absence of ketosis at any time were considered to be the clinical diagnostic criteria for MODY [32].

These criteria are not particularly sensitive or specific, and have been revised on several subsequent occasions. Further research has shown that “MODY” is actually a heterogeneous group of disorders, in that defects in one of several genes may be responsible for the clinical phenotype. As these conditions exhibit significant heterogeneity in their clinical features and management, it has been suggested that lumping them under a single category may be inappropriate. As a consequence, the use of the term MODY is now no longer recommended and the various forms of monogenic diabetes are named based on the gene involved. Some of the better-characterized types of monogenic diabetes are listed in Table 1. According to the International Society for Pediatric & Adolescent Diabetes (ISPAD), a diagnosis of monogenic diabetes cannot be made now without genetic testing [33].

**Table 1:** Different types of monogenic diabetes and the genes implicated [52]

Historical name	Gene	Locus	Clinical features
*MODY 1*	*HNF4A*	20q12–q13.1	Mild-severe fasting and postprandial plasma glucose (PG) Respond well to sulphonylurea agents
*MODY 2*	*GCK*	7p15–p13	Mild fasting hyperglycemia. Less than 50 % of carriers have overt diabetes, and microvascular complications of diabetes are rare. Treatment not needed except in pregnancy (see below)
*MODY 3*	*HNF1A*	12q24.2	Same as MODY 1
*MODY 4*	*IPF1*/ *PDX1*	13q12.1	Pancreatic agenesis.
*MODY 5*	*HNF1B*	17cen–q21.3	Overt diabetes in association with renal and genito-urinary abnormalities.
*MODY 6*	*NEUROD1*	2q32	Rare, with phenotype characterized by obesity and insulin resistance.
*MODY 7*	*KLF11*	2p25	Very rare; phenotype ranges from impaired glucose tolerance or impaired fasting glucose to overt diabetes.
*MODY 8*	*CEL*	9q34.3	Very rare; associated with both exocrine and endocrine pancreatic deficiency and with demyelinating peripheral neuropathy.
*MODY 9*	*PAX4*	7q32	Very rare. Crucial transcription factor for beta cells development
*MODY 10*	*INS*	11p15.5	Very rare. Usually associated with neonatal diabetes. < 1 % cases.
*MODY 11*	*BLK*	8p23–p22	These adapter proteins’ nucleate formation contributes to the qualitative and quantitative control of beta cell signaling.
*MODY 12*	*ABCC8*	11p15.1	Very rare. Usually associated with neonatal diabetes. < 1 % cases.
*MODY 13*	*KCNJ11*	11p15.1	Very rare. Usually associated with neonatal diabetes. < 1 % cases.
*MODY 14*	*WFS*	4p16.1	Rare. Usually associated with DIDMOAD syndrome. Also, seen with early onset diabetes.< 1 % cases.

The enzyme glucokinase controls insulin release from the beta cell by sensing the ambient glucose concentration; inactivation of this enzyme renders the beta cell less capable of responding to hyperglycemia. In patients with heterozygous mutations in the glucokinase gene, the ability to release insulin is not completely lost, and the patient can still respond appropriately to hyperglycemia, albeit at a higher threshold glucose level. These patients therefore present with mildly elevated fasting plasma glucose levels that rise minimally following a glucose load [34]. Individuals carrying the mutation are usually asymptomatic and are detected incidentally. Depending on the age at which the diagnosis is made, these individuals may be mistakenly considered as having T1D, T2D or gestational diabetes. They require no treatment and are not prone to develop complications of diabetes. However, when a woman with a heterozygous GCK mutation becomes pregnant and the fetus does not carry the mutation, there is a risk of development of macrosomia as the fetal pancreas responds appropriately to the elevated maternal glucose levels by increasing its output of insulin. In such cases, treatment of maternal hyperglycemia with insulin is warranted.

Defects in genes responsible for normal pancreatic growth, development, and function (transcription factors, most commonly hepatocyte nuclear factor 1 alpha and hepatocyte nuclear factor 4 -alpha) are responsible for familial young-onset diabetes, historically referred to as MODY 1 and MODY 3. These patients present with progressive hyperglycemia that responds initially to treatment with low dose sulfonylureas, to which they are exquisitely sensitive and respond for prolonged periods of time. Occasionally, progressive beta-cell loss may necessitate initiation of insulin therapy. In the initial stages, these patients may have normal fasting plasma glucose levels, but usually exhibit a steep increase [>5 mmol/l (90 mg/dl)] in glucose levels following an oral glucose load. There is usually a history of early onset diabetes in either parent, although this might have been diagnosed as T1D and treated with insulin [33]. History may also reveal an affected grandparent. These patients are prone to develop micro- and macrovascular complications with the same frequency as T2D.

Certain forms of monogenic diabetes, such as those due to defects in the hepatocyte nuclear factor 1- β (HNF-1 β; historically termed MODY 5), are associated with extra-pancreatic features such as renal cysts. In addition, some rare forms of monogenic diabetes may present as part of well-characterised syndromes, such as the Wolcott-Rallison syndrome and the DIDMOAD (diabetes insipidus, diabetes mellitus, optic atrophy and deafness) syndrome. These do not usually present significant diagnostic challenges.

An interesting variety of monogenic diabetes is Neonatal Diabetes Mellitus (NDM) which is defined as diabetes with onset within the first 6 months of life. Many of these NDM cases are due to defects in the genes encoding the ATP-sensitive potassium channel of the beta cell. Some of these children with NDM can be successfully switched over from insulin injections to oral sulfonylurea tablets after confirmation of the genetic diagnosis [35].

### Diabetes secondary to diseases of the pancreas

#### Fibrocalculous pancreatic diabetes (FCPD)

This is an uncommon form of diabetes secondary to chronic calcific non-alcoholic pancreatitis [36]. It is found most commonly in southern Asia and parts of Africa, but even here, the incidence seems to be falling [37]. The exact etiology is not clear, but genetic and dietary factors have been implicated. The diagnosis is clinical and based on the criteria listed in Table 2 [38].

**Table 2:** Criteria for the diagnosis of FCPD [38]

Diagnostic criteria for FCPD (Mohan et al.)	
1	Patient should be from a tropical country
2	Diabetes should be present
3	Evidence of chronic pancreatitis must be present (abnormal pancreatic morphology on sonography or CT scan, recurrent abdominal pain since childhood, steatorrhea, abnormal pancreatic function tests)
4	Absence of other causes of chronic pancreatitis (alcoholism, hepatobiliary disease, etc.)

Interestingly, although patients with FCPD are insulin deficient and require life-long insulin therapy, they rarely develop diabetic ketoacidosis (DKA). They are prone to develop microvascular complications of diabetes but macrovascular disease is rare. The most dreaded long-term complication is the development of pancreatic carcinoma.

Another, increasingly frequent, form of pancreatic diabetes occurs in children with cystic fibrosis (CF) [39]. Pancreatic damage occurs due to ductal obstruction by abnormally viscid pancreatic secretions, leading to exocrine and ultimately endocrine pancreatic insufficiency. Diabetes occurs in 2 to 3 % of children with CF, and the incidence increases with age. With improvements in long-term survival of patients with CF, the prevalence of diabetes can also be expected to increase. Diabetes is insidious in onset and is chiefly due to beta-cell loss, with a blunted, delayed and prolonged insulin response to a carbohydrate load. Most patients require insulin for control of hyperglycemia. Prolonged survival has also led to an increase in frequency of chronic microvascular complications of diabetes. It has been recommended that all children with CF be screened for diabetes starting at the age of 10 years [40].

### Challenges in diagnosis

#### Clues from history and physical examination

Children and adolescents with diabetes are more likely to present with symptomatic hyperglycemia (polyuria, polydipsia, and weight loss) than are adults, who are often asymptomatic at the time of diagnosis. This may be due to the fact that most pediatric and adolescent cases are linked to T1D, which is a more severe form of the disease, or because children are less likely than adults to have undergone frequent blood testing for unrelated indications, allowing glucose to rise to very high levels before detection ultimately occurs. Nevertheless, the presence or severity of symptoms is not a reliable indicator of the type of diabetes except in the very rare case where isolated fasting hyperglycemia is incidentally detected (glucokinase mutations) or where hyperglycemia occurs in the presence of symptoms of pancreatic insufficiency, such as recurrent abdominal pain and oily stools (FCPD). Initial presentation in DKA usually indicates underlying T1D; however, around 5 % of youth with T2D may also present with DKA at diagnosis (as compared to around 30 % of those with T1D) [41]. In adolescent girls, a history of irregular periods and polycystic ovarian syndrome (PCOS) favors the diagnosis of T2D.

The age of onset is also not a reliable predictor of the type of diabetes. Although T1D is more common in children and adolescents, it can be diagnosed at any age. Unfortunately, incidence of new-onset T1D in those over 20 years of age is unknown. In a study from Europe, adults aged 30–70 years, ∼9 % tested positive for GAD antibodies (GADA) within 5 years of a diabetes diagnosis. This would suggest that T1D is not uncommon among adults, and that they are mostly misdiagnosed as T2D [42]. While T2D is increasingly becoming common at younger ages, onset before puberty remains distinctly unusual [43]. More than 60 % of patients with the hepatocyte nuclear factor 1A (HNF1A) mutation develop diabetes by the age of 25 years, and 80 % by 35 years [33]. FCPD usually develops in the second or third decade of life [38].

**Fig. 1:**
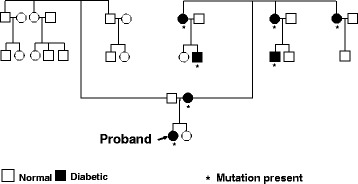
Pedigree chart showing autosomal dominant transmission of diabetes, suggestive of monogenic diabetes

A family history is important to differentiate various types of diabetes. A detailed pedigree chart should be drawn for every patient, covering at least three generations of the family (more if practically feasible). A strongly positive family history of diabetes on goes in favor of a diagnosis of T2D; conversely, T2D is unlikely if neither parent has diabetes. In The SEARCH study, more than 80 % of children and adolescents with T2D had a positive family history of disease [44]. It should, however, be emphasized that a negative family history may also mean that neither parent has ever been screened for diabetes; in such a situation, the parents should be advised to undergo testing as soon as possible. Monogenic forms of diabetes usually follow an autosomal dominant pattern of inheritance, with at least three generations of one side involved, and with at least one member, in addition to the proband, developing diabetes at a young age (Fig. 1). While the risk of developing T1D is 8 to 15-fold higher in first-degree relatives, the majority (>85 %) of newly detected cases are sporadic [45]. A positive family history of diabetes is unusual in patients with FCPD, although familial clustering has been occasionally reported [46].

**Fig. 2:**
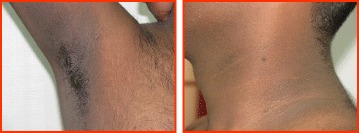
Acanthosis nigricans in a 14-year old boy with T2D Laboratory investigations for differential diagnosis

The physical exam may provide subtle clues to differentiate the type of diabetes. Young individuals with T2D are usually overweight or obese (often morbidly). They usually have markers of insulin resistance (acanthosis nigricans and skin tags). Acanthosis nigricans refers to a velvety hyperpigmented lesion found most often on the nape of the neck and axilla, but also occasionally on the forehead, knuckles, or elbows (Fig. 2). It is not a marker of T2D but only of hyperinsulinemia. It is thought to be due to stimulation of IGF-1 receptors in the dermis, leading to cellular proliferation. It usually regresses partially upon weight reduction and amelioration of insulin resistance. Patients with T1D may be lean, whereas those with monogenic forms of diabetes are usually of normal body weight. It should be noted that with the increase in prevalence of childhood and adolescent obesity, it is no longer unusual for patients with T1D to be overweight or obese [47]. Patients with FCPD are usually lean, and may also show clinical evidence of deficiencies of fat-soluble vitamins A, D, E, and K, as well as essential fatty acids.

The degree of hyperglycemia (as assessed by blood glucose estimation or glycated hemoglobin-HbA1c) does not provide sufficiently reliable indications as to the type of diabetes, although patients with T1D and FCPD tend on average to have higher blood glucose levels than those with T2D. Absence of ketosis in spite of high blood glucose levels in a hitherto untreated patient are suggestive of FCPD, where the concurrent counter regulatory hormone deficiency and the lack of fat stores for ketogenesis provide relative protection against the development of DKA [38].

The lipid profile in T2D may show the classical pattern of “diabetic dyslipidemia”, characterized by high triglycerides, low high density lipoprotein (HDL) cholesterol (HDL-C), and normal or slightly elevated low density lipoprotein (LDL) cholesterol (LDL-C) with a preponderance of highly atherogenic small dense LDL particles. Patients with diabetes due to HNF 1A mutation have low levels of LDL-C and high levels of HDL-C, while the lipid profile in individuals with HNF 4A mutation more commonly resembles that seen in T2D [33].

**Fig. 3:**
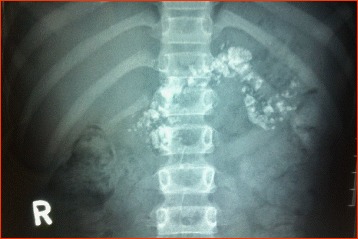
Abdominal X-ray in a patient with FCPD showing pancreatic calculi

Abdominal imaging, by plain x-ray or ultrasonography, is useful in detecting pancreatic pathology in cases of FCPD. Identification of pancreatic calculi in a young patient with diabetes confirms the diagnosis of FCPD (Fig. 3). Ultrasound of the abdomen will also help to delineate the renal and urinary tract pathology characteristic of HNF 1B mutations,, as well as the pancreatic pathology found in several rarer types of monogenic diabetes.

The definitive diagnosis of monogenic diabetes requires genetic testing in patients that qualify for the same owing to the clinical phenotype.

The C-peptide assay is a useful test for identifying the type of diabetes as well as in predicting the response to therapy. The physiological role of C-peptide is to connect the A and B chains of insulin in the proinsulin molecule, which is the immediate precursor of insulin. After cleavage, C-peptide remains in the secretory granules in the beta cell and is released along with insulin in equimolar quantities. Measurement of C-peptide is, therefore, a useful index of endogenous insulin reserve. C-peptide is usually measured in the fasting state and after stimulation, either by means of glucagon administration or ingestion of a standard meal. The reference levels of C-peptide vary based on the individual lab, the method used for estimation, and the stimulus administered. In general, normal or elevated levels of C-peptide are suggestive of T2D; those with monogenic diabetes have intermediate levels and those with FCPD have poor levels, although specific C-peptide cut-points have not been established for the diagnosis of any of these entities. C-peptide levels are profoundly low or absent in patients with T1D, although rarely, residual C-peptide has been detected up to 40 years after diagnosis of T1D [48]. Nonetheless, persistence of detectable C-peptide in a patient with “T1D” after 2 to 3 years of diagnosis should prompt reconsideration of the diagnosis. It should be noted that patients with profound hyperglycemia of any etiology may have suppressed C-peptide levels; this phenomenon is termed “glucotoxicity”. C-peptide estimation is therefore not recommended in the acute phase of hyperglycemia, but after correction of hyperglycemia, C-peptide levels tend to improve, particularly in T2D.

Testing for pancreatic autoantibodies is useful in the diagnosis of T1DM. The antibodies usually looked for include: glutamic acid decarboxylase (GAD), insulin autoantibodies (IAA), insulinoma antigen-2 (IA-2), and zinc transporter-8 (ZnT8). While the presence of high titers of one or more of these autoantibodies makes the diagnosis of T1D likely, a few points must be kept in mind.

- A significant proportion of patients with T1D are autoantibody-negative, particularly in Asia and Africa
- Autoantibody titers tend to diminish with time since onset of diabetes
- Rarely autoantibodies may be positive in T2D

Table 3 shows the differences in clinical presentation and laboratory investigations between the different types of diabetes in youth.

**Table 3:** Clinical and diagnostic features of diabetes in youth

	Type 1 diabetes	Type 2 diabetes	Monogenic diabetes	FCPD
Age at onset	Any age after 6 months; most common in childhood and early adolescence	Adolescence and young adulthood; onset in children becoming more common, although unusual before puberty	Any age; usually presents before 25 years of age; Hyperglycemia in GCK defects can be present from birth	Usually in the 2nd decade
Family history of diabetes	Usually sporadic (>85 %)	Strongly positive; usually on both sides of the family	Positive for at least three generations, on one side of the family	Unusual
Overweight/obesity	Occurs at frequency similar to general population	Common	Occurs at frequency similar to general population	Usually lean
Markers of insulin resistance	Unusual	Common	Unusual	Unusual
C-peptide levels	Low or undetectable, particularly after 2 to 3 years of diagnosis	May be supranormal, normal or low	Usually lower than normal	Low
Islet autoantibodies	Present in majority of patients	Usually absent	Usually absent	Usually absent

**Fig. 4:**
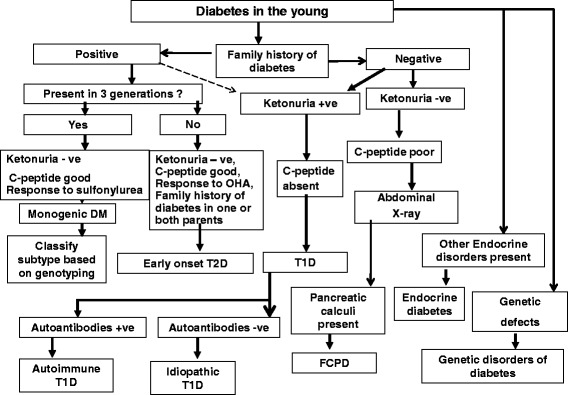
Algorithm for differential diagnosis of diabetes in youth

Figure 4 shows an algorithm for the differential diagnosis of youth-onset diabetes.

### Challenges in management

The implications of a diagnosis of diabetes in childhood and adolescence are profound. Most of the complications of diabetes are related to glycemic control and the duration of disease. An individual diagnosed with diabetes at a young age will invariably be exposed to hyperglycemia for many years resulting in higher risk for diabetes complications. These patients are also likely to develop morbidity and disability due to diabetes complications during the prime of their productive lives. Table 4 details the implications of different types of diabetes in youth.

**Table 4:** Long-term implications of different types of diabetes in youth

Common to all forms of diabetes	Likelihood of prolonged exposure to hyperglycemia Issues with glycemic control during puberty
Type 2 diabetes	Higher prevalence of cardiovascular risk factors
Type 1 diabetes	Risk of diabetic ketoacidosis during intercurrent illness when insulin omitted
Monogenic diabetes due to transcription factor defects	Risk of micro-and macrovascular disease similar to T2D
Monogenic diabetes due to glucokinase defects	Low risk of long-term complications
FCPD	Low risk of macrovascular disease Risk of microvascular disease comparable to T2D

In addition, it has been shown that T2D developing in youth follows a more aggressive course than that with onset later in life [49]. Glycemic control is more difficult, comorbidities are more frequent, and the risk of complications is higher in these individuals compared to those with later-onset disease. Ensuring optimal control of diabetes from the time of diagnosis is therefore essential.

A high index of suspicion should be maintained for the development of chronic complications in all patients with youth-onset diabetes. In T1D, retinopathy and nephropathy are unusual in the first 5 years after onset and screening for these complications is not essential during this time window. In all other forms of diabetes, patients should be screened for complications at the time of diagnosis, on account of the possibility of long-term undiagnosed diabetes [50].

### Management issues in youth onset diabetes

Most patients with T1D would require multiple daily doses of insulin or a continuous subcutaneous insulin infusion (CSII) pump for control of diabetes.

Insulin and metformin are the only agents approved for the management of T2D in children and adolescents [51]. These children also tend to have other cardiovascular risk factors such as hypertension and abnormal lipid profiles, which need treatment according to age-specific guidelines. Lifestyle modification, aimed at preventing further weight gain and loss of excess weight, is essential and will have to be lifelong.

Patients with monogenic diabetes due to defects in transcription factor genes tend to be exquisitely sensitive to low doses of sulfonylureas, at least in the initial stages. A few patients may need insulin as the beta cell defect progresses. As mentioned earlier, diabetes due to heterozygous defects in the glucokinase gene does not require any treatment (the sole exception being pregnant women in whom the fetus shows evidence of growth acceleration) [33].

Most patients with FCPD require insulin injections. Diabetes is usually brittle and difficult to control in these cases; use of a CSII pump may be an attractive option.

## Conclusion

Diabetes is now perhaps the most common endocrine disorder in children and adolescents across the world. Diabetes developing in youth presents several unique challenges: the differential diagnosis is wide, and the clinical course is likely to be more aggressive. Young people with diabetes are more prone to develop disease complications not only because of the long duration of hyperglycemia that they accumulate, but also due to the inherently aggressive nature of the disease and suboptimal control. Unfortunately, the diagnosis of diabetes is often delayed (except in T1D) leading to prolonged periods of uncontrolled hyperglycemia and consequent risk of acute and chronic complications, and misclassification occurs occasionally. Timely and accurate diagnosis, combined with regular follow-up and maintenance of optimal glycemic and risk factor control by judicious use of the available therapies will ensure that these young people enjoy a long, fruitful, and complication-free life in spite of diabetes.

## Abbreviations

CF: Cystic fibrosis
CSII: Continuous subcutaneous insulin infusion
DKA: Diabetic ketoacidosis
FCPD: Fibrocalculous pancreatic diabetes
GAD: Glutamic acid decarboxylase
HbA1c: Glycated hemoglobin
HDL-C: High density lipoprotein (HDL) cholesterol
IA-2: Insulinoma antigen-2
IAA: Insulin autoantibodies
LDL-C: Low density lipoprotein (LDL) cholesterol
MDI: Multiple daily injections
MODY: Maturity onset diabetes of the young
OHA: Oral hypoglycemic agent
PCOS: Polycystic ovarian syndrome
PG: Plasma Glucose
SMBG: Self-monitored blood glucose
T1D: Type 1 diabetes
T2D: Type 2 diabetes
ZnT8: Zinc transporter-8
